# Sexing Adult Pale-Winged Starlings Using Morphometric and Discriminant Function Analysis

**DOI:** 10.1371/journal.pone.0135628

**Published:** 2015-09-14

**Authors:** Laurence Henry, Véronique Biquand, Adrian J. F. K. Craig, Martine Hausberger

**Affiliations:** 1 Université de Rennes 1, UMR CNRS 6552 Ethologie Animale et Humaine, Rennes, France; 2 Department of Zoology & Entomology, Rhodes University, Grahamstown, South Africa; 3 CNRS, Université de Rennes 1, UMR 6552 Ethologie Animale et Humaine, Rennes, France; CNRS, FRANCE

## Abstract

Accurate sexing of birds is vital for behavioral studies but can be a real problem in the field, especially for monomorphic species. Our goal here was to characterize the morphology of male and female monomorphic pale-winged starlings (*Onychognathus nabouroup*), a South African sturnid whose plumage is sexually monomorphic. Morphological measurements of genetically sexed animals indicated that males were statistically larger than females for five measurements: Mass, tail length, tarsus length and wing length. By using a Discriminant Function Analysis based on the measurements taken by one ringer, we were able to predict correctly the sex of 81.10% of the birds of data collected in the field and 77.9% of museum skins independently of year of capture and ringer. The model developed here should be useful for further field studies of this species.

## Introduction

Accurate sexing of birds is a major issue for most ornithological studies but can be a real problem in the field, especially when the species is monomorphic. The only reliable method is to sex birds using genetic information from blood, feather or buccal swab samples after capture, but current techniques are expensive, time consuming [1, 2, 3, 4] and the first two techniques are too invasive to be used for endangered species [5]. This means that critical gender information is not available immediately in the field. One possible approach is to separate males and females by behavioural differences, but this may be impractical in terms of the time available [6] and for species for which precise sex identification is necessary to establish behavioural differences. Morphology can be used for some species when males and females differ in size or mass, but overlap is likely and a multiparameter approach is then required. Discriminant function analysis has been used for an array of species [2] and enables the development of an equation weighting the different parameters according to their relative importance in sex differentiation.

However, sex identification at capture typically requires two steps: 1) a sample of birds of known sex (through DNA or gonadal examination), 2) morphometric measurements of this sample that with an appropriate statistical tool such as a Discriminant Function Analysis, reveal morphological differences.

For DFA to be used for field work, an equation that can characterize birds must be independent of factors such as geographic specificity, temporal variation, sample size and observer [1], [7], [8]. Because phenotypic plasticity can involve morphological traits usually used for biometric calibrations, caution is required when using this method and comparisons with genetic data are desirable [9]. Another potential source of variation is the person taking the measurements because there can be methodological differences or human variability.

Our goal here was to develop a way of sexing pale-winged starlings *Onychognathus nabouroup*, a species from South Africa whose plumage is sexually monomorphic [10], based on morphometric measurements taken in the field. This species is poorly known, and accurate sex determination is crucial for our current studies of their singing behaviour. For instance, birds of both sexes of a congeneric dimorphic species, the Red-winged Starling *Onychognathus morio* sing, but males and females show differences in their song behaviour [11].

We applied a two-step approach to data collected over two seasons of ringing in a population of pale-winged starlings in the Northem Cape in South Africa: 1) Birds were sexed using DNA analyses of feather samples; 2) DFA was applied to the morphological measurements taken when these birds were captured. Four ringers measured birds in the field. Both sexual dimorphism and observer-related differences were investigated, yielding recommendations for using this technique in the field. We validated the models by applying our DFA equation to museum specimens collected in southern Africa and sexed by gonadal examination. Our DFA model provided a high reliability of sex determination based of morphometrics and is potentially a useful tool for field studies of such species.

## Materials and Methods

### Study animals

Pale-winged starlings live in the dry western interior of South Africa, extending northwards through Namibia to the arid coastal strip of south-western Angola. The inner margins of the primary remiges are creamy white, showing a white wing patch in flight. Adult plumage is uniform glossy black, and their iris ranges from bright yellow to orange. Juvenile birds have matt black plumage and a dull-coloured iris. Nest sites and roosting areas are mostly crevices in cliffs, and the birds commonly associate in flocks that forage widely feeding on fruits, nectar, and arthropods. They may scavenge around houses and camp sites.

### Data collection

#### Northern Cape population

In all we caught 65 birds in Augrabies Falls National Park in South Africa from October 26 to October 30 2011 and from October 30 to November 4 2012. Birds are present in the park year-round. They are well habituated to visitors and approach close to chalets and tents to forage. Birds were caught using a flap trap baited with food, triggered by an observer sitting immobile a few meters away. After capture, the birds were immediately measured and ringed with a unique combination of coloured rings as well as a standard, uniquely numbered metal ring. Only qualified ringers recognised by SAFRING (Permit 296) captured and ringed birds. Feather samples were taken under the license number R-2012-MH01 issued by the university animal ethics committee. Handling lasted less than 5 min, and birds were released immediately after having been measured.

Five to ten small breast feathers were removed by ringers and used for genetic sex determination by a veterinary laboratory (Labofarm, France). Forty-two birds were handled in 2011, and 23 in 2012, by four experienced South African ringers (AC, AVZ, MF, PH); 27 by AC (2011), 12 by AVZ (2011), 19 by MF (3 in 2011, 16 in 2012), 6 by PH (2012). The four ringers were at most 200m apart and ringed birds were regularly re-sighted in different parts of the rest camp area. Potential local differences are therefore excluded.

#### Museum sample

This data set included 86 specimens (49 males, 37 females) sexed by gonadal examination, from seven museum collections in different localities in southern Africa collected between 1902 and 1986 (S1 Table).

### Morphological measurements

We took five different morphological measurements for each live bird: body mass (g) using either a Pesola spring balance (0–100g x 1g) with an error of 0.5g (MF) or 1.0 g (AVZ, PH), or an electronic balance with an error of 0.1g (AC). Wing and tail lengths were measured either with a stainless steel rule (AVZ, PH) or a stopped ruler (AC, MF). Tarsus and culmen lengths were measured with Vernier calipers with an accuracy of 0.1mm. All ringers measured wing length using the flattened chord method. Tail length was measured from the base of the tail to the tip of the longest tail feather. Culmen length was measured either from the bill tip to the base of the skull (AVZ) or from the bill tip to the feathering (AC, MF and PH). Tarsus length was measured from the joint to the last complete scale above the foot, which corresponds to the length of the tarso-metatarsal bone [12].

Four measurements were taken from all museum specimens by AC: Wing, tarsus, culmen (to the base of the skull) and tail length. Body mass was not recorded for any of these specimens.

### Statistical analyses

An ANOVA was performed assessed differences between males and females, between individual ringers, and potential interaction effects between ringer and sex of the bird. Two further analyses were then performed on the data collected by the ringer who had measured the largest sample of birds (AC: N = 27, 10 males and 17 females): 1) t tests compared males and females and examined sexual dimorphism [13]. 2) Discriminant function analyses (DFA) were performed to construct a model to predict a bird's sex on the basis of morphological measurements. Using data from a single ringer eliminated one source of variation (ringer effect). This model was then validated using the data collected by other ringers in 2011 and 2012. To generalize the method, we constructed a second model based on all 2011 and 2012 data. Both models were then tested using data from museum specimens sexed by dissection, measured by the same person (AC). These two DFA models were validated using the cross validation method. All statistical tests were performed using R software and the MDA, Mass, KlaR and Boot library. The level of significance was set as p < 0.05. Means are given ± SD.

## Results

The DNA analyses revealed that 31 of the 65 birds caught in Augrabies were males and 34 were females (47.7% / 52.3%). Sex ratio did not vary significantly between the two years (40.5% / 59.5% in 2011, 60.9% / 39.1% in 2012, Chi² test: χ² = 2.48, df = 3, p< 0.05).

Morphological data (Table 1)

**Table 1:** Morphological characteristics for males and females.

Measurements	MalesN = 31mean±SD(range)	FemalesN = 34mean±SD(range)	pt test
Mass (g)	103.55±5.83(89.60–114.00)	96.72±7.02(83.50–109.00)	0.00006 *
Wing (mm)	144.99±3.77(134.5–150.00)	138.80±3.76(131.00–146.00)	0.00000001 *
Tail (mm)	106.85±4.80101.00–117.00)	102.48±4.97(92.00–117.00)	0.0004 *
Tarsus (mm)	31.85±1.23(28.50–34.20)	30.20±1.01(27.80–32.30)	0.0000002 *
Culmen (mm)	21.43±2.11(18.30–27.60)	20.32±2.09(17.5–23.80)	0.03 *

All data. SD: standard deviation

*: significant difference.

**Fig 1:**
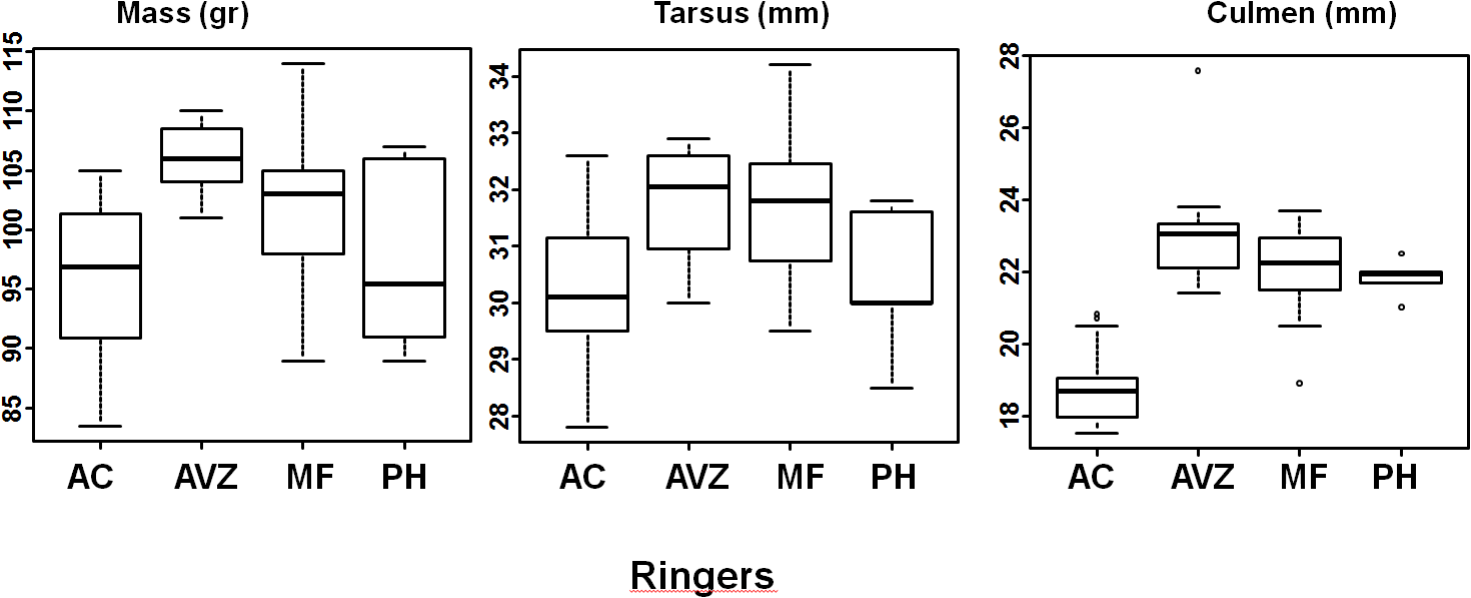
Differences of morphological measurements between ringers. Box plots of mass, tarsus and culmen data recorded by the four ringers over two years.

The ANOVA performed on the data for birds caught in Augrabies revealed both sex and ringer effects for three of the variables: mass, tail length and tarsus length (Anova: mass: sex: F = 18.08, df = 1, p< 0.001, ringer: F = 8.49, df = 3, p<0.001; tail length: sex: F = 22.04, df = 1, p<0.001, ringer: F = 6.00, df = 3, p< 0.001; tarsus length: sex: F = 33.97, df = 1, p< 0.001, ringer: F = 8.24, df = 3, p< 0.001), a ringer effect for culmen length (Anova: F = 42.40, df = 3, p< 0.001) and a sex effect (Anova: F = 42.56, df = 1, p< 0.001) as well as an interaction effect between sex and ringers (Anova: F = 3.51, df = 3, p< 0.05) for wing length (Fig 1). On average, males were heavier than females (Males: 103.55±5.83 range: 89.60–114.00: Females: 96.72±7.02, range: 83.5–109), had longer wings (Males: 144.99±3.77 range: 138.00–150.00, Females: 138.80±3.76, range: 131.00–146.00), longer tails (Males: 106.85±4.30 range: 101.00–117.00, Females: 102.48±4.97, range: 92.00–117.00), longer tarsi (Males: 31.85±1.23 range: 28.50–34.20, Females: 30.20±1.01, range: 27.80–32.30) and longer culmens (Males: 21.22±1.80 range: 18.30–23.70, Females: 20.32±2.09, range: 17.50–23.80) (Table 1).

We found no significant differences or interaction effects between years (Anova, P >0.05 in all cases) thus data from both years were pooled for further analyses. Some measurements were correlated: mass was correlated with tarsus, wing and culmen length (Spearman rank correlation rs = 0.6, N = 65, p = < 0.001, rs = 0.58, N = 65, p < 0.001 rs = 0.57, N = 65, p < 0.001, tail and wing length were correlated (rs = 0.51, N = 65, p< 0.001, tarsus length was correlated to wing and culmen length (rs = 0.65, N = 65, p < 0.001, rs = 0.54, N = 65, p <0.001), and wing length was correlated with culmen length (rs = 0.37, N = 65, p < 0.01). Tail length was not correlated with any of the other parameters.

**Fig 2:**
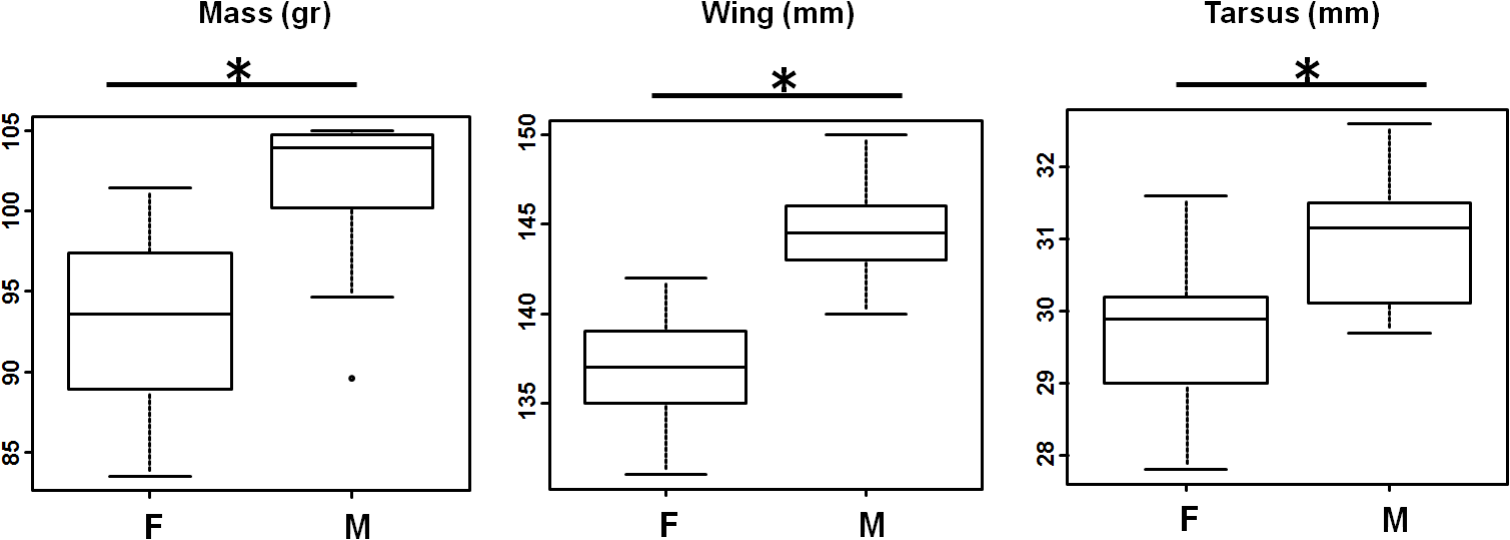
Morphological measurement differences between males and females. Box plots of mass, wing and tarsus data. All data were collected by a single ringer (AC). *: significant difference at p ≤ 0.05.

T tests on the data collected by the main ringer confirmed these findings (Fig 2). We found significant differences between males and females for four parameters (mass, tail, wing and tarsus length). Culmen length did not differ between sexes (Table 2).

**Table 2:** Morphological characteristics for males and females.

Measurements	MalesN = 10mean±SD(range)	FemalesN = 17mean±SD(range)	pt test
Mass (g)	101.29±5.24(89.60–104.70)	93.11±5.09(83.50–101.40)	0.0009 *
Wing (mm)	144.45±2.85(140.00–150.00)	136.74±3.11(131.00–142.00)	0.000002 *
Tail (mm)	110.80±4.55(103.00–117.00)	104.25±5.40(97.00–117.00)	0.003 *
Tarsus (mm)	31.06±0.90(29.70–32.60)	29.74±1.00(27.80–31.60)	0.002 *
Culmen (mm)	18.98±0.85(18.30–20.80)	18.55±0.96(17.5–20.70)	>0.05 NS

All measurements were taken by a single ringer (AC). SD: standard deviation; NS: non-significant

*: significant difference.

### Sex differences and the ringer effect

As mentioned above, the ANOVA revealed a ringer effect for most measurements. When comparing the data recorded by the two ringers who had measured the most birds (AC and MF), we found significant differences between them for all measurements as well as a sex effect for mass, tail, tarsus and wing (Anova: mass: ringer: F = 6.64, df = 1, p < 0.05, sex F = 21.3, df = 1, p = 3.4510^−5^; tail: ringer: F = 7.71, df = 1, p< 0.01, sex, F = 17.55, df = 1, p< 0.001; tars: ringer, F = 17.23, df = 1, p< 0.001, sex: F = 32.41, df = 1, p< 0.001; wing: ringer: F = 13.82, df = 1, p < 0.001, sex, F = 59.84, df = 1, p< 0.001; culmen: ringer: F = 113.99, df = 1, p< 0.001, sex: F = 2.54, df = 1, p> 0.05). Average mass differed for males (3.96g) and for females (4.83g), mean wing lengths differed for males (2.47mm) and for females (4.26mm), tail lengths differed for males (4.6mm) and for females (2.68mm), tarsus lengths differed for males (1.51mm) and for females (0.87mm) and culmen lengths difference for males (3.35mm) and for females (3.2mm) between the two ringers. These differences were statistically significant for most measurements (Anova: males: tail, F = 9.68, df = 1, p< 0.01, tarsus: F = 13.97, df = 1, p < 0.001, culmen: F = 90.1, df = 1, p, < 0.001). Thus, morphological measurements should be taken by a single individual for reliable sexing of birds in the field.

### Predicting sex by discriminant analysis

#### Using one ringer’s data (AC)

A step by step DFA revealed that three variables (wing, tail and tarsus) were relevant to discriminate males from females. The combination of these three variables gave the best rate of discrimination possible taking into account sample size and type of variable measured. A linear model gave better results than a binomial model. The discriminant function was:

D=−24.72+0.14(wing)+0.01(tail)+0.16(tarsus), Eq 1

If D>0.5, birds were classified as male and if D<0.5, birds were classified as female. A cross validation analysis using the same set of birds indicated that this model allowed us to predict the sex of 92.3% of the birds. Only one male and one female were misclassified.

When this model was applied to MF's data (19 birds measured), sex could be accurately predicted for 89.5% of these birds (17/19). Again, only one male and one female were misclassified, with 11 (of 12) males (91.7%) and 6 (of 7) females (87.5%) correctly classified. When we added data from the two other ringers (AVZ: 12 birds, PH: 6 birds), collected in 2011 and 2012 (N = 38), sex was correctly assigned to 81.1% of the birds: 17 males (of 21: 80.95%) and 14 females (of 17: 82.35%). Misclassified males were small and misclassified females were large.

In order to test this model further, we applied it to the museum data set: 86 birds measured by AC. We were able to predict the sex of 77.9% of the birds, but 11 males and 8 females were misclassified. Therefore 70% of the males were correctly classified (26 / 37) and so were 83.67% of the females (41 / 49). We assume of course that these birds were sexed correctly on collection. These specimens came from a wide geographical area, including Angola, Namibia, and South Africa (S1 Table).

#### Using the whole sample

In a second step, we completed a Discriminant Function Analysis on data collected on all birds in Augrabies in 2011 and 2012 with the same step by step method. Again, the same three variables, wing, tail and tarsus lengths, appeared as the most relevant variables for discriminating males and females. 85.8% of the birds were correctly sexed with only 4 / 32 (12.5%) females and 5 / 31 (16.1%) males incorrectly sexed. We obtained the following discriminant function:

D=−21,65+0.059(Wing)+0.047(Tail)+0.27(Tarsus), Eq 2

If D>0.5, birds were classified as male and if D<0.5, birds were classified as females.

When applying this second equation to the museum set of data, we predicted correctly the sex of 76.74% of the birds. As for the preceding analysis, 26 males (of 37, 70.3%) were correctly classified and 40 females (of 49: 81.63%) were correctly classified. This second equation therefore performed slightly worse, misclassifying 20 birds, than the first equation that misclassified 19. The two models misclassified the same 15 birds, whereas the first model misclassified 4 different birds and the second model 5 other birds. No clear pattern of errors emerged as misclassified specimens came from 16 different localities.

## Discussion

Our results show that male and female pale-winged starlings can be separated using simple morphological parameters. Males are on average heavier than females and have longer wings, tails and tarsi. Using discriminant function analysis (Eq 1), we determined the sex accurately of at least 81.1% of birds in the field and 77.9% of the museum skins, independently of year of capture and ringer by measurements. Our further investigation revealed that sex determination using a model could predict the sex of up to 92.3% of the birds when the same person took all the measurements. The fact that we found a significant ringer effect calls for caution when comparing measurements obtained from different sources.

### Differences between males and females

Thus this apparently monomorphic species [10] presents a clear morphological sexual dimorphism. The morphological parameters used in this study were those most commonly used for characterizing birds. Body mass, wing and tarsus lengths as well as head and bill characteristics are the parameters usually measured. Two to 12 variables are usually incorporated into models, but whatever number used, this still fails to separate males from females for some species. Seven of ten variables discriminated significantly long-tailed finch (*Poephila acuticauda*) males from females [14], whereas the nine variables measured were all useful to discriminate Humboldt penguins’ (*Spheniscus humboldti*) sex [6]. Van de Pol et al. (2009) found just four variables that all differentiated oystercatcher (*Haematopus ostralegus*) males from females. Thus, variables that differentiate males from females for a given species may not be critical for another species. For example, body mass differences distinguish significantly between Humboldt penguin males and females [6] but not between bay-capped wren-spinetails (*Spartonoica maluroides*) [5]. Subtle differences between males and females can be found in many species. Therefore a species cannot be wisely categorized as strictly monomorphic before detailed investigation [14]. Several studies show that species known as sexually monomorphic do in fact present differences between sexes: long tailed finches (*Poephila acuticauda*) [14], bay-capped wren-spinetails [5], northem shrikes (*Lanius excubitor*) [15] and Acadian flycatchers (*Empidonax virescens*) [16] for example. Birds may use features such as ultraviolet pigmentation of plumage to discriminate sexes [17, 18]. Monomorphic or monochromatic classifications may therefore in many cases be more a human-based classification than a biological reality [19]. Another intriguing point is that some dimorphic individuals may have the appearance of one sex but belong to the other sex as for instance rose-colored starlings (*Sturnus roseus*) [20], and represents another potential area of misidentification. In the European starling (*Sturnus vulgaris)* and in the wattled starling (*Creatophora cinerea*) older females may present male characteristics [21, 22].

### DFA as a tool for distinguishing males from females

Apart from theoretical considerations, not being able to distinguish males from females remains a real problem in fieldwork. Behavioural cues are not always reliable [1] and same sex individuals (usually females) may associate together as social pairs either outside the breeding period when males’ and females’ plumages differ less (European starlings, [23]) or when sex ratios are biased during the breeding season (roseate terns, *Sterna dougalii* [24]).

When males and females are apparently similar, animals have to be individually marked for long-term behavioural observations. This means that the animals have to be caught at least once, thus providing an opportunity either to measure morphological parameters, or to take buccal swabs, feather or blood samples for DNA analyses. DNA analysis appears to be the most accurate method and is essential for birds to obtain a reference set of individuals of known sex [25]. However, it is costly and moreover does not allow immediate identification, thus delaying the start of a behavioural study after capture; therefore to have an immediate sexing method is crucial. To overcome this problem, standardized morphological measurements when compared to genetic identification, enable the establishment of a mathematical equation using DFA that can be used in the field. Although different problems may emerge (a range of uncertainty, see next paragraph), this method can avoid using expensive time consuming techniques repeatedly [1, 2].

Here, we were able to determine reliably the sex of at least 81.1% of birds from a second set of data including measurements made by different ringers on live birds (Eq 1). This percentage falls within the range reported by other authors (from 63% to 100%, see [2] for a review). Our sample was small in comparison to those of most studies using morphometric measurements but it represented the largest sample ever collected for this species. We did not obtain 100% certainty of sex, but this is only a first step and we hope to refine the model as more birds are ringed in future. We were not able to link misclassifications to precise features. Data indicated that geographical origin of birds was not involved but some males are particularly small whereas some females are particularly large. Therefore a certain range of uncertainty exists. However, errors of sexing may possibly occur using DNA sexing [25].

### Observer effect, a recurrent concern?

One striking finding of this study was the ringer effect that obviously can limit the accuracy of sex determination. One way to reduce this bias would be to verify systemically that all observers use exactly the same method for measurements. Clearly caution is required as variations of morphological measures are often reported and ascribed to different factors. Differences may occur between populations (black terns [1]; cape petrels [8]), years or season (oystercatchers [9]), or between captive or wild animals [6] and as in our case, between observers [16]. Several of these studies present measurements from different sets of data collected by different observers (e.g. [6], [8]), and this alone could explain some of the variation. Measurement variations can be due to different factors. Ringers do not use exactly the same tools (some could be more precise than others) and do not have the same training. Moreover humans’ accuracy can potentially vary. DFA, as a multiparameter tool, enabled us to overcome this problem and this lead to high rate of inter-observer reliability. The model developed here (Eq 1) should therefore be useful for further studies of this species.

## Supporting Information

S1 Table Information concerning museum specimen: source, specimen number, sex of individuals, date and locality where birds were found. (PDF)
