# Supplementary material for: Sexing Adult Pale-Winged Starlings Using Morphometric and Discriminant Function Analysis
Source: PLoS One. 2015 Sep 14;10(9):e0135628. doi: 10.1371/journal.pone.0135628 (PMC4569554; doi:10.1371/journal.pone.0135628)
Supplement: S1 Table — (PDF) [file pone.0135628.s001.pdf]

Supplementary data concerning museum specimen

Source	**Specimen number**	**Sex**	**Date**	**Locality**
Ditsong (formerly Transvaal) Museum, Pretoria, South Africa	TM 24381	M	01 July 1941	Augrabies, South Africa
Ditsong (formerly Transvaal) Museum, Pretoria, South Africa	TM 4691	M	01 October 1917	Klawer, South Africa
Ditsong (formerly Transvaal) Museum, Pretoria, South Africa	TM 2417	F	01 August 1912	Philipstown, South Africa
Ditsong (formerly Transvaal) Museum, Pretoria, South Africa	TM 1502	F	01 April 1910	Middelburg, South Africa
American Museum of Natural History, New York, USA	AMNH669225	M	20 May 1902	Deelfontein, South Africa
American Museum of Natural History, New York, USA	AMNH260078	M	01 May 1925	Lobito Bay, Angola
American Museum of Natural History, New York, USA	AMNH260079	F	02 May 1905	Lobito Bay, Angola
American Museum of Natural History, New York, USA	AMNH388536	M	14 December 1953	Kaiser Wilhelm Mt, Namibia
American Museum of Natural History, New York, USA	AMNH388537	M	15 December 1953	Okahandja, Namibia
American Museum of Natural History, New York, USA	AMNH388538	M	28 December 1953	Okahandja, Namibia
American Museum of Natural History, New York, USA	AMNH423661	F	22 July 1936	Otjosongombe, Namibia
American Museum of Natural History, New York, USA	AMNH423662	F	14 July 1936	Otjosongombe, Namibia
Windhoek Museum, Namibia	CA 3819	F	17 October 1970	Swartbooisdrift, Namibia
Windhoek Museum, Namibia	CA 2914	M	18 March 1968	Komashochland, Namibia
Windhoek Museum, Namibia	CA 3443	F	10 October 1968	Warm Quelle, Namibia
Windhoek Museum, Namibia	CA 2788	M	13 July 1967	Garib, Namibia
Windhoek Museum, Namibia	CA 2996	M	18 May 1968	Waterberg, Namibia
Windhoek Museum, Namibia	CA 4273a	F	27 September 1974	Bethanien, Namibia
Windhoek Museum, Namibia	CA 4273b	M	01 October 1974	Bethanien, Namibia
Windhoek Museum, Namibia	CA 4273c	F	30 September 1974	Bethanien, Namibia
Windhoek Museum, Namibia	CA 4273d	F	30 September 1974	Bethanien, Namibia
Windhoek Museum, Namibia	CA 4274a	F	08 October 1974	Bethanien, Namibia
Windhoek Museum, Namibia	CA 4274b	F	07 October 1974	Bethanien, Namibia
Windhoek Museum, Namibia	CA 4274c	M	04 October 1974	Bethanien, Namibia
Windhoek Museum, Namibia	CA 4274d	F	04 October 1974	Bethanien, Namibia
Windhoek Museum, Namibia	CA 4275a	F	15 October 1974	Karasburg, Namibia
Windhoek Museum, Namibia	CA 4275b	M	13 October 1974	Karasburg, Namibia
Windhoek Museum, Namibia	CA 4275c	M	15 October 1974	Karasburg, Namibia

Windhoek Museum, Namibia	CA 4275d	M	15 October 1974	Karasburg, Namibia
Windhoek Museum, Namibia	CA 4275e	M	15 October 1974	Karasburg, Namibia
Museum Alexander Koenig, Bonn, Germany	MAK 1936.113	F	23 April 1935	Otjosongombe, Namibia
Museum Alexander Koenig, Bonn, Germany	MAK 1939.55	M	19 November 1938	Naukluft, Namibia
Museum Alexander Koenig, Bonn, Germany	MAK 1948.91	F	27 August 1947	Karibib, Namibia
Museum Alexander Koenig, Bonn, Germany	MAK 1939.89	M	02 January 1939	Karibib, Namibia
Museum Alexander Koenig, Bonn, Germany	MAK 1934.317	F	09 June 1934	Otjosongombe, Namibia
Museum Alexander Koenig, Bonn, Germany	MAK 1939.54	F	28 November 1938	Marienthal, Namibia
Museum Alexander Koenig, Bonn, Germany	MAK 1934.316	M	25 April 1934	Otjosongombe, Namibia
Museum Alexander Koenig, Bonn, Germany	MAK 1966.22	F	16 November 1965	Huila, Angola
Smithsonian Institution, Washington DC, USA	USNM 484219	F	19 November 1963	Gobabeb, Namibia
Smithsonian Institution, Washington DC, USA	USNM 331152	F	15 January 1931	Brukkaros, Namibia
Smithsonian Institution, Washington DC, USA	USNM 263811	M	05 November 1916	Cradock, South Africa
Albany Museum,Grahamstown, South Africa	AM 3638	M	15 January 1986	Klein Pella, South Africa
Albany Museum,Grahamstown, South Africa	AM 3639	F	15 January 1986	Klein Pella, South Africa
Albany Museum,Grahamstown, South Africa	AM 3640	M	16 January 1986	Klein Pella, South Africa
Albany Museum,Grahamstown, South Africa	AM 3641	M	16 January 1986	Klein Pella, South Africa
Albany Museum,Grahamstown, South Africa	AM 3642	F	16 January 1986	Klein Pella, South Africa
Albany Museum,Grahamstown, South Africa	AM 3643	F	16 January 1986	Klein Pella, South Africa
Natural History Museum, Tring, UK	BM1965M18805	F	12 June 1949	Carnarvon, South Africa
Natural History Museum, Tring, UK	BM1850.50.1306	F	07 January 1950	Kleinkaras, Namibia
Natural History Museum, Tring, UK	BM1904.6.20.1	F	24 July 1902	Klipfontein, South Africa
Natural History Museum, Tring, UK	BM1905.12.29.588	F	17 April 1903	Klipfontein, South Africa
Natural History Museum, Tring, UK	BM1905.12.29.593	F	05 June 1903	Klipfontein, South Africa
Natural History Museum, Tring, UK	BM1905.12.29.591	F	04 May 1903	Klipfontein, South Africa
Natural History Museum, Tring, UK	BM1903.3.9.715	F	20 May 1902	Deelfontein, South Africa
Natural History Museum, Tring, UK	BM1903.3.9.712	F	15 April 1902	Deelfontein, South Africa
Natural History Museum, Tring, UK	BM1901.9.5.74	M	12 January 1901	Deelfontein, South Africa
Natural History Museum, Tring, UK	BM1903.3.9.713	M	23 April 1902	Deelfontein, South Africa
Natural History Museum, Tring, UK	BM1903.3.9.710	M	26 March 1902	Deelfontein, South Africa
Natural History Museum, Tring, UK	BM1903.3.9.711	M	23 March 1902	Deelfontein, South Africa
Natural History Museum, Tring, UK	BM1903.3.9.718	M	22 December 1902	Deelfontein, South Africa
Natural History Museum, Tring, UK	BM1903.3.9.717	M	22 December 1902	Deelfontein, South Africa

Natural History Museum, Tring, UK	BM1903.3.9.716	M	12 June 1902	Deelfontein, South Africa
Natural History Museum, Tring, UK	BM1928.10.25.70	M	04 December 1926	Cradock, South Africa
Natural History Museum, Tring, UK	BM1904.7.23.3	M	27 February 1904	Kuruman, South Africa
Natural History Museum, Tring, UK	BM1950.50.1305	M	07 January 1951	Kleinkaras, Namibia
Natural History Museum, Tring, UK	BM1950.50.1307	M	08 January 1951	Kleinkaras, Namibia
Natural History Museum, Tring, UK	BM1905.12.29.589	M	02 May 1903	Klipfontein, South Africa
Natural History Museum, Tring, UK	BM1950.50.1308	M	10 February 1950	Helmeringshausen, Namibia
Natural History Museum, Tring, UK	BM1905.12.29.590	M	04 May 1903	Klipfontein, South Africa
Natural History Museum, Tring, UK	BM1905.12.29.592	M	06 May 1903	Klipfontein, South Africa
Natural History Museum, Tring, UK	BM1950.50.1304	M	23 December 1949	Vioolsdrift, Namibia
Natural History Museum, Tring, UK	BM1905.12.29.587	M	14 April 1903	Klipfontein, South Africa
Natural History Museum, Tring, UK	BM1903.3.9.714	M	19 May 1902	Deelfontein, South Africa
Natural History Museum, Tring, UK	BM1965M18808	M	27 May 1949	Karibib, Namibia
Natural History Museum, Tring, UK	BM1965M18804	M	02 June 1949	Keetmanshoop, Namibia
Natural History Museum, Tring, UK	BM1965M18806	M	30 April 1949	Calvinia, South Africa
Natural History Museum, Tring, UK	BM1928.10.25.69	M	04 December 1926	Cradock, South Africa
Natural History Museum, Tring, UK	BM1906.12.4.287	M	07 September 1905	Benguella, Angola
Natural History Museum, Tring, UK	BM1957.35.526	F	14 September 1957	Novo Redondo, Angola
Natural History Museum, Tring, UK	BM1906.12.4.285	F	07 September 1905	Benguella, Angola
Natural History Museum, Tring, UK	BM1906.12.4.286	M	12 September 1905	Damaraland, Namibia
Natural History Museum, Tring, UK	BM1936.7.19.42	M	12 February 1936	Otjosongombe, Namibia
Natural History Museum, Tring, UK	BM1936.7.19.43	F	12 February 1936	Otjosongombe, Namibia
Natural History Museum, Tring, UK	BM1934.2.12.8	M	09 December 1933	Naukluft, Namibia
Natural History Museum, Tring, UK	BM1904.7.23.4	F	27 February 1904	Kuruman, South Africa
Natural History Museum, Tring, UK	BM1965.m.18807	F	07 June 1949	Prieska, South Africa
